# Euler simulation of hydrocyclone pre-separation before oily wastewater membrane treatment based on Population balance model

**DOI:** 10.1038/s41598-026-45695-8

**Published:** 2026-03-24

**Authors:** Zhao Shuai, Xie Liqiu, Ding Laiyuan, Liu Yan, Sun Daming, Ren Yong

**Affiliations:** Shandong Labor Vocational and Technical College, Jinan, 250300 China

**Keywords:** Oily wastewater, Membrane separation pretreatment, Hydrocyclone separator, Oil-water separation, Population balance model, Energy science and technology, Engineering, Environmental sciences, Mathematics and computing

## Abstract

Membrane technology is considered an effective technique for separating and purifying oily wastewater, and membrane fouling caused by surfactant adsorption or oil droplet blockage of pore channels. Therefore, before membrane treatment of oilfield wastewater, it is necessary to carry out pretreatment to reduce the oil content of oilfield wastewater and lay the foundation for improving membrane treatment efficiency. This article mainly uses the finite volume method to discretize the control equations, and uses the pressure velocity coupling method to analyze the primary hydrocyclone separation efficiency of oilfield produced fluids with oil content ranging from 10 to 30%. It explores the hydrocyclone flow field and particle trajectory of mixed fluids in hydrocyclones under different oil content states. The results indicate that the distribution of oil phase volume fraction in the hydrocyclone is comprehensively controlled by inlet flow velocity, oil content, and structural parameters such as cone angle and overflow pipe depth. At low to medium flow velocities (5.0 ~ 15.0 m/s), the centrifugal force field dominates the enrichment of oil towards the axis, and the coalescence effect is enhanced. At high inlet flow rates, the oil phase outflow channel changes from a dual channel of overflow and bottom flow to mainly overflow. When the inlet flow rate reaches 20 m/s, the shear force increases, causing the oil droplets to break and the radial migration path to deviate from the design. The flow velocity distribution becomes more complex, resulting in the distribution characteristics of the oil volume fraction in the mixed liquid becoming more complex, and small-sized oil droplets are discharged from the bottom flow. The increase in oil content enhances the probability of coalescence by increasing viscosity, but also increases Stokes resistance, resulting in prolonged droplet retention time, increased risk of shear failure, and intensified fluctuations in oil phase volume fraction at the overflow port.

## Introduction

With the deepening popularization of environmental protection, energy conservation, and efficiency concepts worldwide, the efficiency and economy of petroleum processing equipment are facing increasingly stringent requirements. In the field of oilfield exploitation, China’s main oilfields have generally entered the stage of medium to high water content exploitation, and with the continuous expansion of the application scale of polymer flooding technology, the production of polymer containing wastewater is increasing year by year^1^. This phenomenon not only directly increases the operating costs of oilfield development, but also significantly increases the technical difficulty of surface separation processes due to the high viscosity of polymer containing wastewater, thereby causing a dual negative impact on the overall economy of development^2,3^. The deep treatment and resource utilization of oily wastewater are of great significance for solving water scarcity, protecting the ecological environment, serving energy conservation and emission reduction, and achieving the “dual carbon” goals.

Membrane technology, as a strategy for separating and purifying oily wastewater, has significant advantages of high efficiency and low energy consumption, and is considered one of the most effective technologies for treating oily wastewater. Through pore size screening and surface wettability control, this technology can achieve selective separation of oil-water mixtures. Xie et al. designed a hyperbranched polylysine grafted PVDF oil-water separation membrane, which showed low adhesion during emulsion oil-water separation, especially under acidic or alkaline conditions, and exhibited ultra-low shear friction adhesion testing in tangential underwater oil, with a separation efficiency exceeding 90%^4^. Jiang et al. constructed a unique layered hydrophilic composite membrane. For the n-hexane oil in water lotion with a flux of 5423 L/(m²‧h‧bar), the separation efficiency is > 99.2%. When treating actual oilfield wastewater, the composite membrane maintained a separation efficiency of 84% after 5 cycles^5^. Wu et al. prepared four types of hydrophobic nanocellulose using freeze-drying method and surface modified them with methyltrimethoxysilane (MTMS) to enhance oil removal efficiency. In the experiment, the aerogel also showed excellent adsorption capacity for various oils and organic solvents, up to 112.30 g/g, and maintained an absorption capacity of more than 74% after five cycles^6^. Zakariyah A. Jamiu et al. proposed a polyethersulfone (PES) film modified by layered chalcogenides (especially germanium sulfide (GeS) and titanium disulfide (TiS)) functionalized with polydopamine (PDA) to treat low interfacial tension and high stability oil in water lotion. The results showed that the modified membrane exhibited excellent oil repellency − 99.9%, and also achieved a high-purity water flux of 7200 LMH/bar and a flux recovery rate of 94.8%^7^. However, membrane fouling caused by surfactant adsorption or oil droplet blockage of pores remains one of the biggest challenges in the treatment of oily wastewater^8,9^. Surface oil pollution can lead to a decrease in flux and separation efficiency of oil-water separation membranes, as well as an increase in operating energy consumption^10^. Therefore, before membrane treatment of oilfield wastewater, it is necessary to carry out pretreatment to reduce the oil content of oilfield wastewater, laying the foundation for saving membrane treatment costs and improving membrane treatment efficiency.

As an efficient solid-liquid separation equipment, hydrocyclones are widely used in coal mines, petroleum, chemical, environmental protection and other fields due to their advantages of simple operation, small footprint, and low maintenance costs^11^ In this context, it is a significant opportunity for liquid-liquid hydrocyclones, but at the same time, it faces enormous challenges. How to further improve the separation efficiency of hydrocyclones and deepen their efficient operational performance is an important issue that urgently needs to be addressed^12^. Jing and Arjun Kumar Pukkella et al. optimized the structure of the hydrocyclone and explored the effects of lubrication components and surface roughness on the separation performance of the hydrocyclone^13,14^. Zhang and Liu et al. studied the effects of the number of inlet ports and structural parameters of the hydrocyclone on separation efficiency and split ratio^15–17^. Li and Li et al. optimized the structure of the hydrocyclone center and bottom flow port, and used a combination of experimental and numerical simulation methods to investigate the influence of hydrocyclone oil-water separation performance^18,19^. Some researchers have also attempted to investigate the influence of process parameters and medium types on oil-water separation efficiency during the operation of hydrocyclones^20,21^. LIU et al. used the Reynolds Stress Model (RSM) and Multiphase Flow Model (Mixture) to study the effect of liquid inlet on the separation characteristics of a gas lift co outlet hydrocyclone. The results showed that as the liquid inlet increased, the volume fraction of the oil phase at the center of the hydrocyclone increased significantly, and the separation efficiency of the hydrocyclone increased from 64% to 77.9%^22^. Wang et al. found through experimental comparison that, the traditional axial hydrocyclone separator separationof 65%, through the ordinary bubble assisted separation efficiency of 74%, and through the preparation of condensate bubbles to enhance flotation can be raised to 89% of the oil removal^23^. Zhao et al. studied the effect of lipophilic particles on the oil-water separation efficiency of hydrocyclones, and the results showed that adding lipophilic particles can effectively adsorb and transport oil droplets, significantly improving the oil-water separation efficiency, with a separation efficiency of up to 99.97%^24^.In addition, considering the mechanism of hydrocyclone separation, the ability to achieve efficient separation of small particle size dispersed phases is a major challenge that restricts the deep improvement of hydrocyclone separation efficiency. Huang et al. developed a new model to predict the separation efficiency of oil droplets in a hydraulic hydrocyclone based on the principle of oil droplet mass balance, based on the analysis of flow patterns, droplet dynamics, and oil concentration distribution. The feasibility of the model was verified through experiments^25^. Joowan Kim et al. developed a parallel small-scale hydraulic cyclone system, which analyzed the variation of vortices with feed flow rate by directly visualizing the primary and secondary vortices generated within the device^26^. On the basis of considering droplet size change and population balance model, Te Bu et al. studied the insertion angle and radius of the 10 mm micro hydrocyclone inlet through numerical simulation, so as to improve the emulsification performance of water in oil and oil in water lotion^27^.

In summary, researchers have attempted to predict the swirl flow field, discrete phase separation efficiency, and particle trajectory using VOF model, Mixture model, DPM model, and particle population equilibrium PBM model^28^. However, the oilfield produced fluid is a complex mixed fluid system, and neither membrane separation technology nor hydrocyclone separation process can complete the oil-water separation process of the mixed fluid in one go. The research team of the author considers that membrane separation has a small processing capacity and is prone to oil phase pollution and adhesion blockage of oil-water separation membranes. Therefore, they propose the idea of membrane separation of the mixed liquid after reducing the oil content of the mixed liquid based on oil-water separation of the oilfield produced liquid. In order to achieve efficient and stable operation of oil-water separation membranes, this paper mainly uses the finite volume method to discretize the control equations, and uses the pressure velocity coupling method to analyze the primary hydrocyclone separation efficiency of oilfield produced fluids with oil content ranging from 10% to 30%. It explores the hydrocyclone flow field and particle trajectory of mixed fluids in hydrocyclones under different oil content states, and predicts the discrete phase separation efficiency, laying the foundation for membrane separation technology of oily mixtures and reducing the problem of oil droplet blockage in the membrane separation process, in order to achieve deep treatment and resource utilization of oily wastewater.

## Mathematical physical models

### Model parameters

**Fig. 1:**
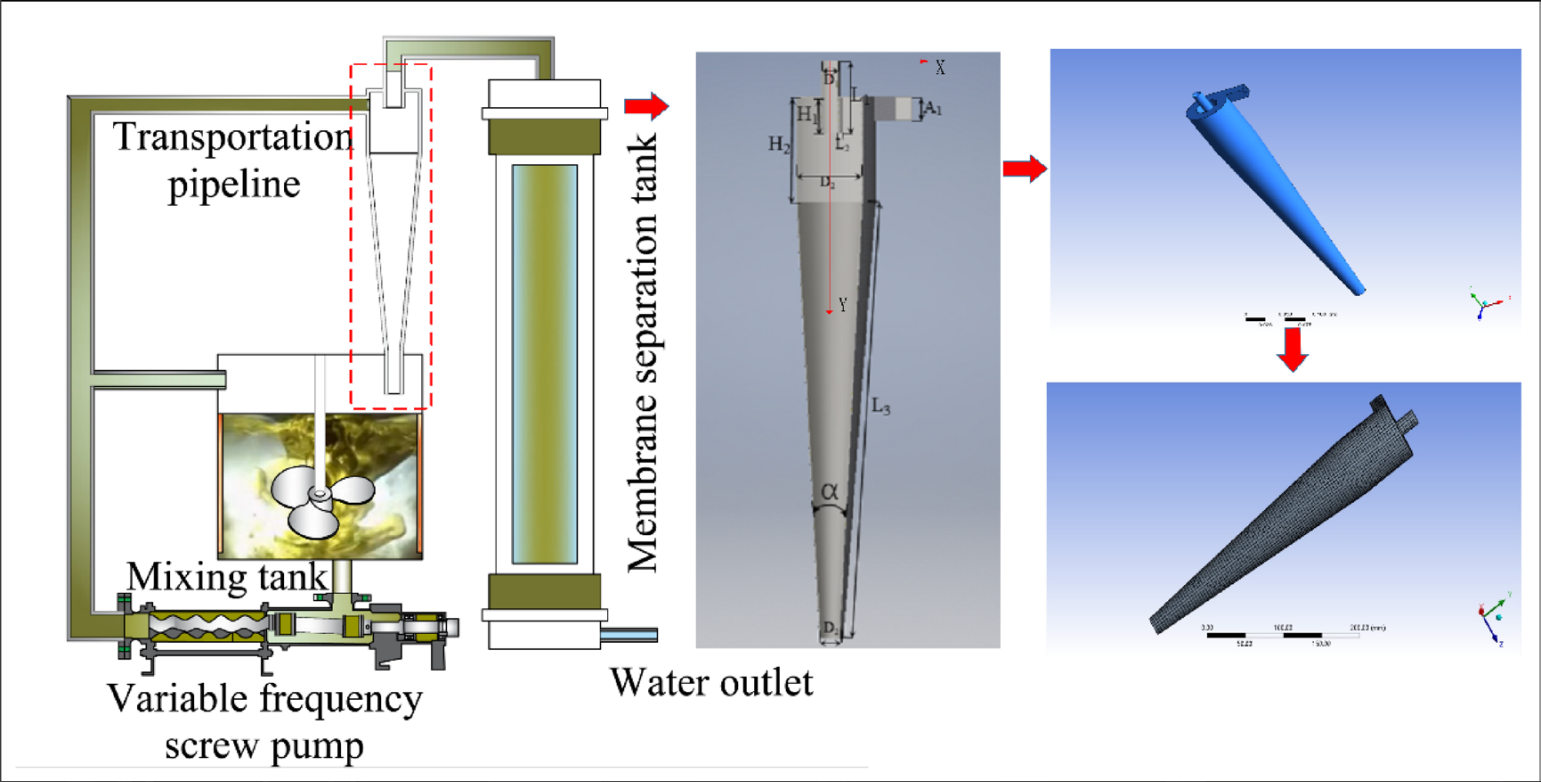
Model structure and grid division results.

The model selected in this article is a tangential single inlet single cone hydrocyclone for the pretreatment of oily wastewater. The hydrocyclone consists of five parts: inlet pipe, cylindrical section, conical section, overflow pipe, and bottom outlet. The specific structural parameters are shown in Table 1. The 3D model construction of the hydrocyclone is completed through Inventor. Using Fluent pre-processing program Workbench to partition hexahedral structured grids, as shown in Fig. 1. Due to the significant difference in fluid flow performance between the near wall region and the turbulent region, in order to improve the accuracy of simulation analysis, the standard wall function method is selected, and this method is combined with the pressure drop at the inlet and outlet of the hydrocyclone to verify the model grid independence. The final selected grid cell size is 2.2 mm, and the boundary layer is defined as a shell non slip impermeable boundary with a thickness of 0.8 mm.

**Table 1:** Structural parameters of tangential single inlet single cone hydrocyclone.

Attribute	Symbol	Numerical value
Entrance cross-sectional area	A_1_	125.00mm^2^
Overflow pipe diameter	D_1_	4.00 mm
Overflow pipe length	L_1_	20.00 mm
Depth of overflow pipe	H_1_	10.00 mm
Interval between overflow pipe and cylindrical section	L_2_	6.25 mm
Diameter of cylindrical segment	D_2_	50.00 mm
Cylinder segment height	H_2_	70.00 mm
Cone segment length	L_3_	360.00 mm
Taper angle	α	11.75°
Bottom outlet diameter	D_3_	12.00 mm

### Model settings

#### Numerical simulation methods and boundary conditions

The simulation calculation uses a dual precision pressure based algorithm with an implicit solver for steady-state solution. The Reynolds Stress Model (RSM) is selected as the turbulence calculation model. The Mixture model is selected for multiphase flow, where the water phase is the continuous phase and the oil phase is the discrete phase. The specific parameters are shown in Table 2. Considering that oil droplets may undergo coalescence and fragmentation under the action of strong swirling flow fields, a coupled particle swarm equilibrium PBM model is proposed. Select Turbulent model for the coalescence model and Luo model for the fragmentation model, and describe the multiphase flow motion in the vortex field. Discretize the control equations using the finite volume method, use the QUICK method for volume fraction, and use the PRESSO! For pressure term! method. The volume fraction of oil phase in the Mixture model is 10%~30%. The inlet of the model is velocity inlet, with an inlet flow velocity of 5.0 ~ 20.0 m/s, and the outlet is free outflow. The convergence of the calculation is based on the fact that the imbalance error of each phase flow rate within a unit time of import and export is less than 10^− 3^.

**Table 2:** Characteristic parameters of oil-gas two-phase steam.

Attribute	Symbol	Numerical value
Density of discrete phase oil droplets	*ρ*	889.2 kg/m^3^
Heat transfer coefficient of oil	*k*	0.14 W/(m·K)
Viscosity of oil phase	*µ*	3.320$\:\times\:$10^−3^Pa‧s
Density of main phase water	*ρ*_*2*_	988.2 kg/m^3^
Heat transfer coefficient of water	*k*_*2*_	0.59 W/(m·K)
Viscosity of water phase	*µ*_*2*_	1.003$\:\times\:$10^−3^ Pa‧s
Average particle size of oil droplets	*d*	100 ~ 200 μm
Oil content		10%;20%;30%
Inlet velocity	*u*	5;10;15;20 m/s

#### Reynolds stress model (RSM)

It is crucial to accurately simulate the fluid flow inside the hydrocyclone when the fluid is in a highly turbulent state in the hydrocyclone field. The Reynolds Stress Model (RSM) is a fundamental tool for engineering turbulence calculations. RSM strictly considers factors such as streamline bending, vortices, rotation, and rapid changes in tension, making it more suitable for rotating flows with strong turbulent transport anisotropy. Considering the high viscosity of crude oil, the efficiency of oil-water separation under conventional conditions is relatively poor. Therefore, team members tested the oily wastewater used in an experiment from a certain oilfield in the Bohai region of China, and measured an oil content of 9.47–17.64% and an oil phase density of 0.924 g/cm^3^. The viscosity of the oil phase measured at different temperatures is shown in Table 3^29^.

**Table 3:** Characteristics of viscosity variation of oil phase in a certain oilfield with temperature.

Temperature /℃	Viscosity /mpa·s	Density /kg/m^3^
0	107 ~ 113	924
20	68 ~ 74	913
45	33 ~ 38	906
70	27 ~ 31	903

In order to reduce the influence of crude oil viscosity on oil-water separation efficiency, the crude oil temperature selected in the numerical simulation process was 70 ℃. Among them, the transport equations for each component of RSM are^29^:

1 $$\frac{{\partial \left( {\rho \overline{{u_{i}^{'} u_{j}^{'} }} } \right)}}{{\partial t}} + \frac{{\partial \left( {\rho u_{k} \overline{{u_{i}^{'} u_{j}^{'} }} } \right)}}{{\partial x_{k} }} = \frac{{D\left( {\rho \overline{{u_{i}^{'} u_{j}^{'} }} } \right)}}{{Dt}} = D_{{i,j}} + P_{{i,j}} + G_{{i,j}} + \Phi _{{i,j}} - \varepsilon _{{i,j}} + F_{{i,j}}$$

In the formula,

D_i, j_ represents the diffusion term of the turbulence model;

P_i, j_ represents the term for shear stress generation;

G_i, j_ represent buoyancy generation terms;

φ_i, j_ represents pressure strain redistribution;

ε_i, j_ represents the viscous dissipation term;

F_i, j_ represent two types of system rotation terms.

2 $$\:{D}_{i,j}=-\frac{\partial\:}{\partial\:t}\left[\rho\:\stackrel{-}{{u}_{i}^{{\prime\:}}{u}_{j}^{{\prime\:}}{u}_{k}^{{\prime\:}}}+\stackrel{-}{P\left({\delta\:}_{kj}{u}_{i}^{{\prime\:}}+{\delta\:}_{ik}{u}_{j}^{{\prime\:}}\right)}-\mu\:\frac{\partial\:}{\partial\:{x}_{k}}\left(\stackrel{-}{{u}_{i}^{{\prime\:}}{u}_{j}^{{\prime\:}}}\right)\right]$$

3 $$\:{P}_{i,j}=-\rho\:\left(\stackrel{-}{{u}_{i}^{{\prime\:}}{u}_{k}^{{\prime\:}}}\frac{\partial\:\stackrel{-}{{u}_{j}^{{\prime\:}}}}{\partial\:{x}_{k}}+\stackrel{-}{{u}_{j}^{{\prime\:}}{u}_{k}^{{\prime\:}}}\frac{\partial\:\stackrel{-}{{u}_{i}^{{\prime\:}}}}{\partial\:{x}_{k}}\right)$$

4 $$\:{G}_{i,j}=-\rho\:\beta\:\left({g}_{i}\stackrel{-}{{u}_{j}^{{\prime\:}}\theta\:}+{g}_{j}\stackrel{-}{{u}_{i}^{{\prime\:}}\theta\:}\right)$$

5 $$\:{\varPhi\:}_{i,j}=\stackrel{-}{P\left(\frac{\partial\:{u}_{j}^{{\prime\:}}}{\partial\:{x}_{i}}+\frac{\partial\:{u}_{i}^{{\prime\:}}}{\partial\:{x}_{j}}\right)}$$

6 $$\varepsilon _{{i,j}} = 2\mu \overline{{\frac{{\partial u_{i}^{'} \partial u_{j}^{'} }}{{\partial x_{k} \partial x_{k} }}}}$$

7 $$F_{{i,j}} = - 2\rho \Omega _{x} \left( {\overline{{u_{j}^{'} u_{m}^{'} }} \varepsilon _{{ikm}} + \overline{{u_{i}^{'} u_{m}^{'} }} \varepsilon _{{jkm}} } \right)$$

In the formula,

β is the coefficient of thermal expansion, m/K;

Ω is the vortex vector;

θ is the cone angle of the hydrocyclone, °;

g is the acceleration due to gravity, 9.81 m/s^2^;

Substitute D_i, j_, P_i, j_, G_i, j_, φ_i, j_, ε_i, j_, and F_i, j_ into the RSM transport Eq. (1) for calculation and simplification. The turbulent kinetic energy equation and turbulent kinetic energy dissipation equation are as follows,

Used for boundaries:

8 $$\frac{\partial }{{\partial t}}\left( {\rho k} \right) + \frac{\partial }{{\partial x_{i} }}\left( {\rho ku_{i} } \right) = \frac{\partial }{{\partial x_{j} }}\left[ {\left( {\mu + \frac{{\mu _{i} }}{{\sigma _{k} }}} \right)\frac{{\partial k}}{{\partial x_{j} }}} \right] + \frac{1}{2}\left( {P_{{i,j}} + G_{{i,j}} } \right) - \rho \varepsilon \left( {1 + 2M_{t}^{2} } \right) + S_{k}$$

Used for hydrocyclones in other watersheds:

9 $$\frac{\partial }{{\partial t}}\left( {\rho \varepsilon } \right) + \frac{\partial }{{\partial x_{i} }}\left( {\rho \varepsilon u_{i} } \right) = \frac{\partial }{{\partial x_{j} }}\left[ {\left( {\mu + \frac{{\mu _{i} }}{{\sigma _{k} }}} \right)\frac{{\partial \varepsilon }}{{\partial x_{j} }}} \right] + 0.72\left( {P_{{i,j}} + C_{\varepsilon } G_{{i,j}} } \right)\frac{\varepsilon }{k} - 1.92\rho \frac{{\varepsilon ^{2} }}{k} + S_{k} ~$$

Where,

*ρ* is the density, kg/m^3^;

*P* is pressure, Pa;

*k* is the thermal conductivity, W/m^2^· K;

*µ* is the fluid dynamic viscosity, N·s/m^2^;

*u*_*i*_ is the velocity vector;

C_ɛ_ is the coefficients related to convection in the k - ε equation. When the fluid direction is parallel to the direction of gravity, C_ɛ_=1. When the fluid direction is perpendicular to the direction of gravity, C_ɛ_=0.

#### Population balance model (PBM)

The Population balance model (PBM) describes the redistribution of dispersed phase oil droplet particle size in mixed liquid systems in research^31^. By considering the particle size changes caused by coalescence and fragmentation between particles, it mainly describes the balance of particles by adding an equilibrium equation based on momentum and energy conservation. The conservation equation of the community balance model is,

10 $$\frac{\partial }{{\partial t}}\left( {n\left( {V,t} \right)} \right) + \nabla \cdot \left[ {\overset{\lower0.5em\hbox{$\smash{\scriptscriptstyle\rightharpoonup}$}} {u} *n\left( {V,t} \right)} \right] = S\left( {V,t} \right)$$

In the formula, *V* is the volume of the droplet; $$\:T$$ is time; $$\overset{\lower0.5em\hbox{$\smash{\scriptscriptstyle\rightharpoonup}$}} {u}$$is the liquid flow; *N* is the quantity density; *S(V*,* t)* is the source term for droplet coalescence and fragmentation, which can be specifically expressed as,

11 $$\:S\left(V,t\right)={B}_{C}\left(V,t\right)-{D}_{C}\left(V,t\right)+{B}_{B}\left(V,t\right)-{D}_{B}\left(V,t\right)$$

12 $$\:{B}_{C}\left(V,t\right)=\frac{1}{2}{\int\:}_{0}^{\infty\:}a(V-{V}^{{\prime\:}},{V}^{{\prime\:}})n\left(V-{V}^{{\prime\:}},t\right)n\left({V}^{{\prime\:}},t\right)d{V}^{{\prime\:}}$$

13 $$\:{D}_{C}\left(V,t\right)={\int\:}_{0}^{\infty\:}a\left(V,{V}^{{\prime\:}}\right)n\left(V,t\right)n\left({V}^{{\prime\:}},t\right)d{V}^{{\prime\:}}$$

14 $$\:{B}_{B}\left(V,t\right)={\int\:}_{0}^{\infty\:}\rho\:g({V}^{{\prime\:}})\beta\:\left(V|{V}^{{\prime\:}}\right)n\left({V}^{{\prime\:}},t\right)d{V}^{{\prime\:}}$$

15 $$\:{D}_{B}\left(V,t\right)=g\left(V\right)n(V,t)$$

In the formula, *ɑ* is the coalescence frequency; *B*_*C*_ *(V*,* t)* and *D*_*C*_ *(V*,* t)* are the droplet generation and fragmentation caused by coalescence, respectively; *B*_*B*_*(V*,* t)* and *D*_*B*_*(V*,* t)* represent droplet generation and fragmentation caused by fragmentation, respectively.

In this model, the Turbulent coalescence model divides the particle coalescence mechanism into viscous coalescence and inertial coalescence based on the relationship between particle diameter and minimum vortex size, and calculates them independently. The Hamaker constant is set to a default value of 2.3 × 10^− 20^. The Luo fragmentation model is a particle fragmentation rate model based on isotropic uniform turbulence theory and probability statistics. It has strong advantages in calculating particle fragmentation rate and sub particle size distribution function, with a surface tension coefficient of 0.0728 N/m.

## Results and discussion

The CFD numerical simulation method based on community balance model (CFD-PBM) was used to numerically simulate the dynamic separation process of oil droplet coalescence and fragmentation in a conventional tangential inlet single cone hydrocyclone. The oil containing wastewater studied in this article has a volume concentration range of 10% to 30% and an operating flow rate of 5.0 to 20.0 m/s. The hydrocyclone used in this article has a cone angle of 11.75 ° and an overflow pipe insertion depth of 100 mm. The influence of factors such as inlet flow rate, particle size distribution, and oil phase viscosity on the flow field distribution characteristics and oil-water separation characteristics of hydrocyclones was discussed.

### Flow velocity distribution characteristics

**Fig. 2:**
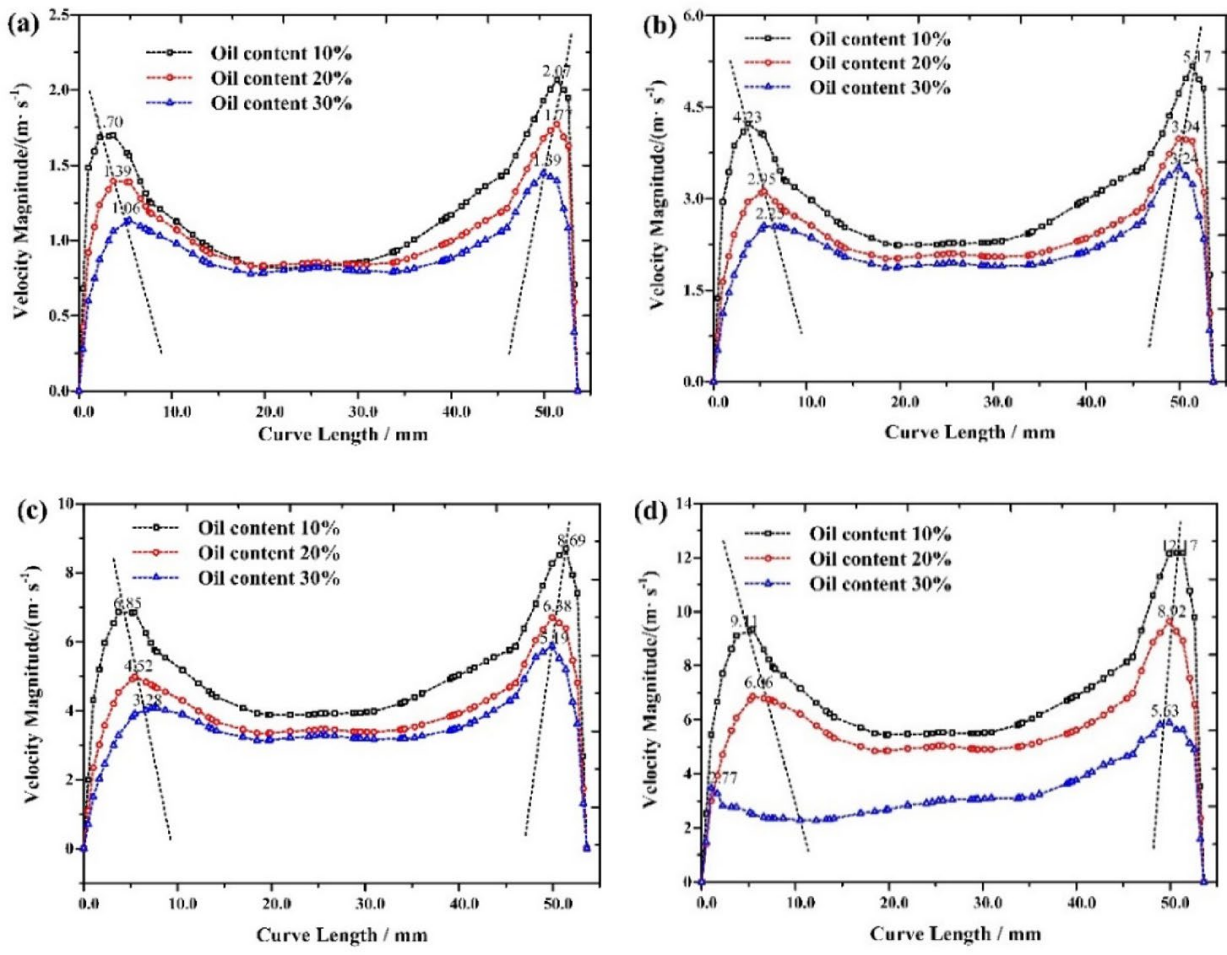
Radial velocity distribution of swirl chamber with different oil content in mixed liquid. (**a**) inlet velocity = 5.0 m/s, (**b**) inlet velocity = 10.0 m/s, (**c**) inlet velocity = 15.0 m/s, (**d**) inlet velocity=20.0 m/s.

**Fig. 3:**
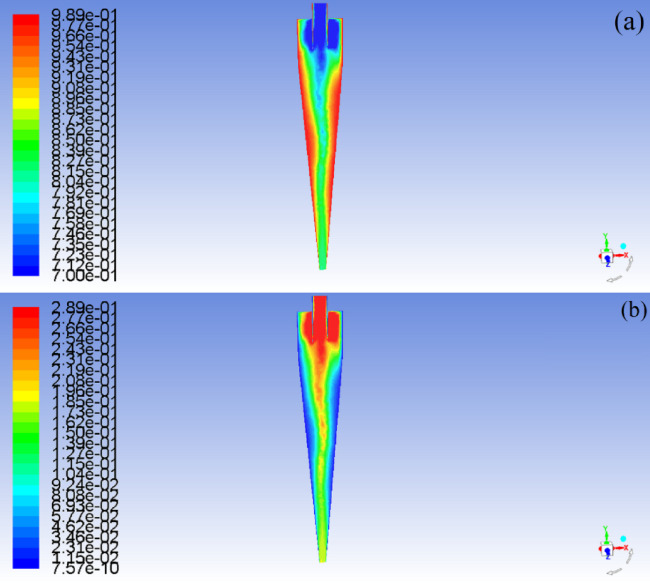
Phase distribution of the mixed liquid in the hydrocyclone at an inlet flow of 15.0 m/s with an oil content of 10%. (**a**) Distribution of water phase (**b**) Distribution of oil phase.

Set up monitoring curves at Y=-70 and X=-25 in the swirl chamber to extract the distribution characteristics of radial flow velocity. The results are shown in Fig. 2, and the overall radial flow velocity presents an M-shape. The radial velocity near the wall of the swirl chamber in the free vortex zone is higher than that in the forced vortex zone. Whether in the forced vortex zone or the free vortex zone, the radial velocity of the mixed liquid in the swirl chamber is lower than the inlet velocity when the inlet velocity is 5.0 m/s ~ 20.0 m/s. The radial flow velocity determines the motion trajectory of oil droplets in a mixed liquid in a centrifugal field. When the fluid enters the swirl chamber tangentially, the centrifugal force generated causes the continuous phase water particles to move towards the wall, while the dispersed phase oil particles gather towards the center, as shown in Fig. 3^30^. At the same inlet flow rate, as shown in Fig. 2a, an increase in the oil content of the mixed liquid leads to a decrease in radial flow velocity. When the oil content of the mixed liquid is 10%, the maximum radial flow velocity in the swirl chamber is 2.07 m/s. When the oil content of the mixed liquid increases to 30%, the maximum radial flow velocity in the swirl chamber decreases to 1.59 m/s. This trend remains consistent when the inlet flow rate of the mixed liquid is between 5.0 m/s and 15.0 m/s. In addition, when the inlet flow velocity of the mixed liquid reaches 20 m/s, the increase in oil content of the mixed liquid leads to an increase in shear force in the near wall area^31^, and the fragmentation phenomenon is more significant. At this point, the relationship between the radial migration velocity and the balance between centrifugal force and drag force is disrupted, and some oil droplet trajectories deviate from the design path. As the radial flow velocity decreases, a more complex distribution trend is presented, as shown in Fig. 2d.

**Fig. 4:**
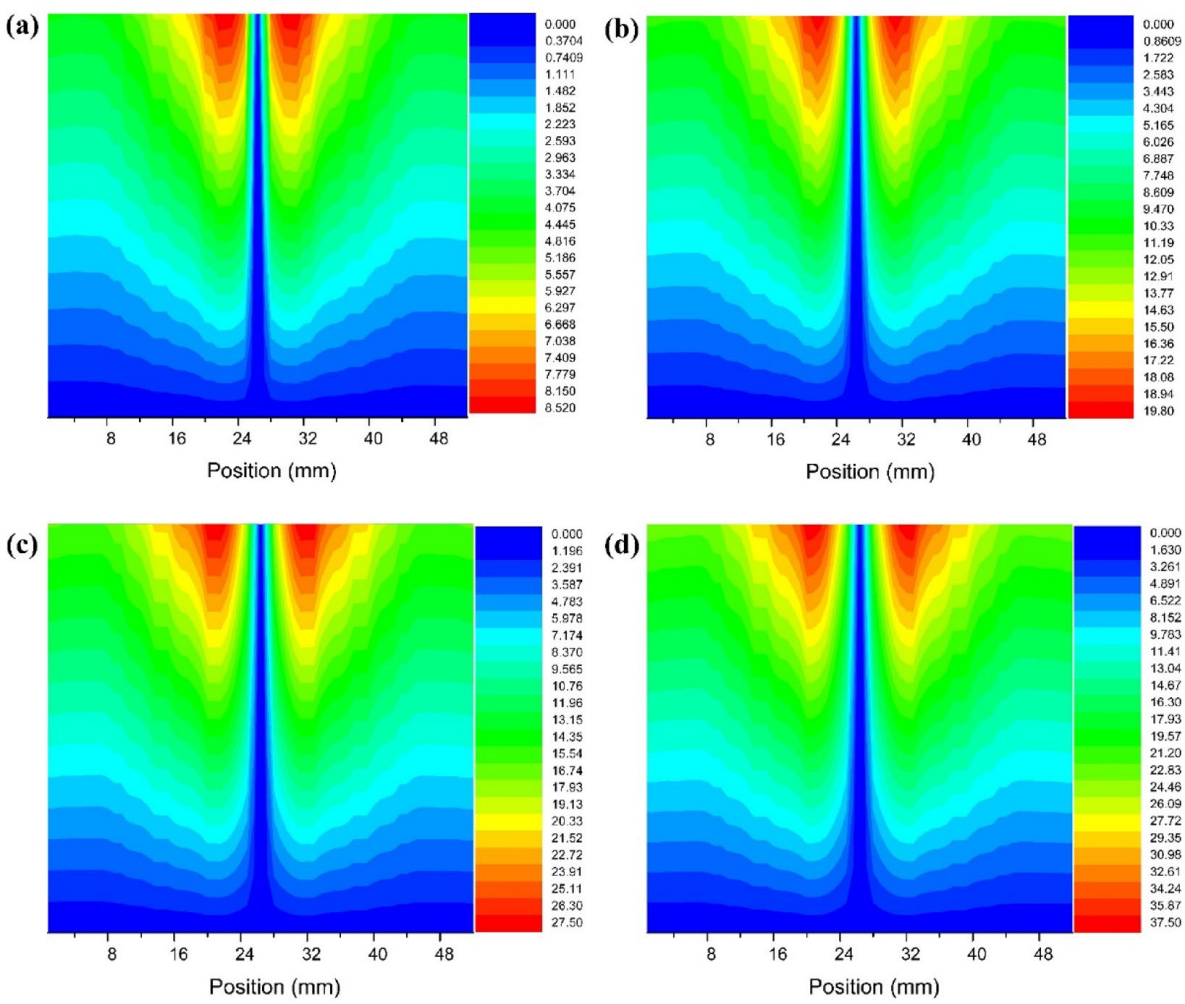
Distribution characteristics of tangential flow velocity in the cone section of the hydrocyclone (**a**) inlet velocity = 5.0 m/s (**b**) inlet velocity = 10.0 m/s (**c**) inlet velocity = 15.0 m/s. (**d**) inlet velocity = 20.0 m/s.

Monitoring curves were set up at Y=-270 and X=-25 in the cone section to extract the distribution characteristics of tangential flow velocity. The results are shown in Fig. 4. The distribution of tangential flow velocity in the cone section is symmetrical along the axis of the cone section, and the tangential flow velocity in the forced vortex zone increases with the increase of radius. When the inlet flow velocity of the mixed liquid is 5.0 ~ 20.0 m/s, the maximum tangential flow velocity in the cone section is about 1.75 ~ 1.87 times that of the inlet flow velocity of the mixed liquid. The result obtained by Li et al. is about 1.5 times, which is mainly related to the angle setting of the hydrocyclone cone section and the viscosity of the mixed liquid^32^. When the radial size is close to the overflow port size, the tangential flow velocity reaches its maximum value. In the free vortex region, the tangential flow velocity shows a decreasing trend with the increase of radius, reaching its minimum value at the cone wall, which is consistent with the conclusion obtained by Ren et al.^33^. After the mixed liquid enters the cone section in a tangential direction, the tangential flow velocity increases compared to the inlet flow velocity and the tangential flow velocity in the swirl chamber. And when the inlet velocity of the mixed liquid increases from 5.0 m/s to 20.0 m/s, the tangential velocity of the cone section also increases with the increase of the inlet velocity. In summary, the influence of droplet coalescence and fragmentation on the separation performance of hydrocyclones was studied using the CFD-PBM numerical simulation method. The obtained tangential velocity distribution characteristics are consistent with those obtained by Jia^31^ and Li^34^ in their research on oil-water cyclone separation.

### Radial pressure distribution characteristics

**Fig. 5:**
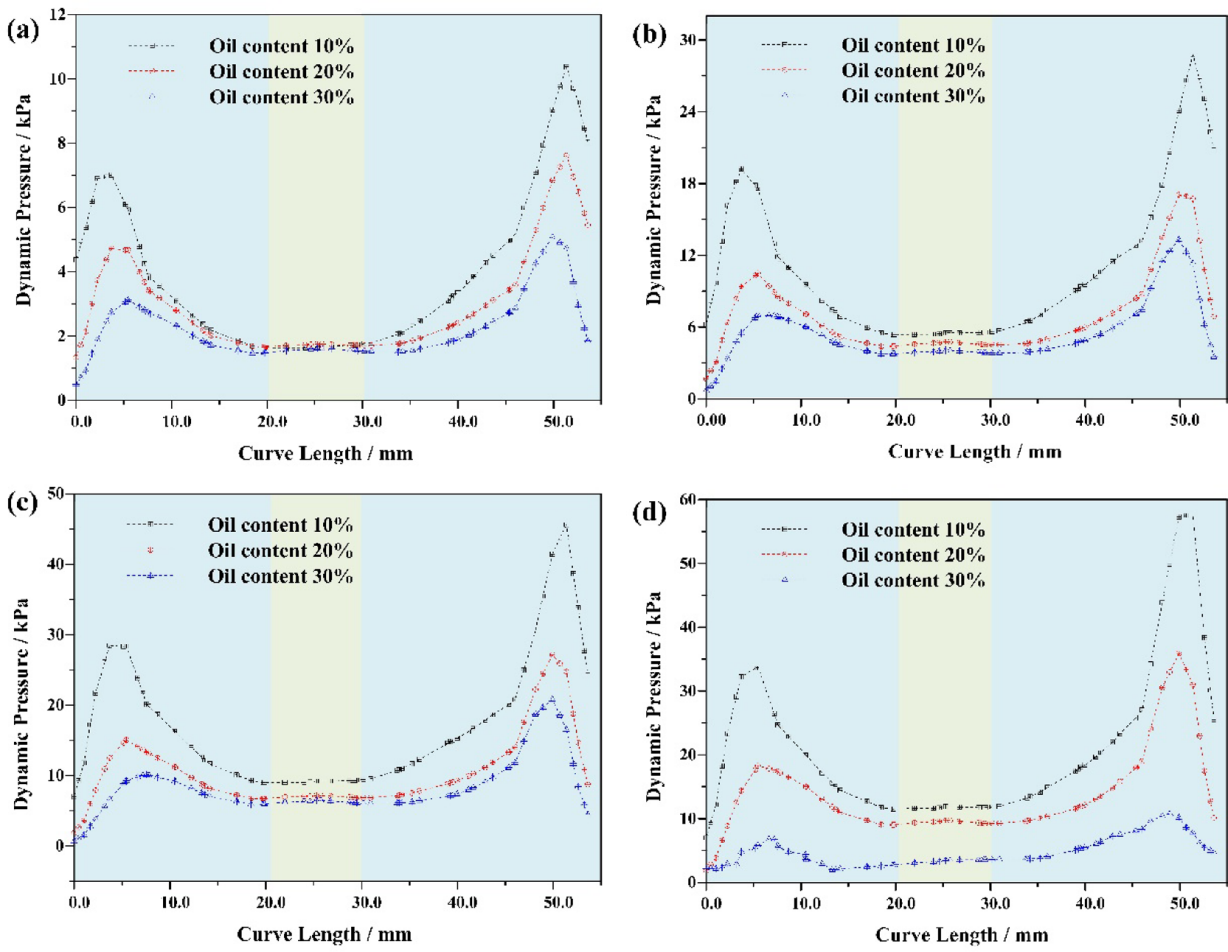
Radial pressure distribution characteristics of hydrocyclone cone section with different oil content in mixed liquid (**a**) inlet velocity = 5.0 m/s (**b**) inlet velocity = 10.0 m/s (**c**) inlet velocity = 15.0 m/s (**d**) inlet velocity = 20.0 m/s.

Radial pressure is the manifestation of the centrifugal force generated by the rotation of the mixed liquid in the radial direction, and the balance with the force on the oil phase particles determines the position of the separation interface, that is, the swept range of the forced vortex zone. Its distribution directly affects the separation trajectory of oil phase particles^15,35^. Set monitoring curves at Y=-270 and X=-25 in the cone section to extract the distribution characteristics of radial pressure in the cone section, as shown in Fig. 5. The distribution trend of radial pressure in the cone section is basically the same under different inlet flow velocities; The radial pressure is higher at the wall of the hydrocyclone than at the axis. The radial migration force F of oil phase particles is mainly determined by the centrifugal force *Fn* experienced by the oil phase particles, the radial force *Fp* generated by the radial pressure difference, and the Stokes Resistance *Fs*^2,36^.

Among them,

16 $$\:F={F}_{P}-{F}_{n}-{F}_{s}$$

17 $$\:{F}_{P}=\frac{\pi\:}{6}{d}^{3}{\rho\:}_{w}\frac{{v}_{t}^{2}}{r}$$

18 $$\:{F}_{n}=\frac{\pi\:}{6}{d}^{3}{\rho\:}_{o}\frac{{v}_{t}^{2}}{r}$$

19 $$\:{F}_{s}=3\pi\:\mu\:d{v}_{r}$$

**Fig. 6:**
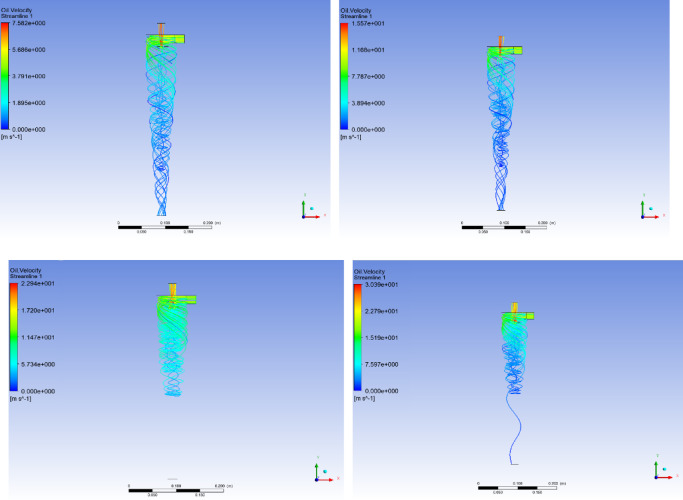
Flow line distribution characteristics of oil phase medium in hydrocyclone. (**a**) inlet velocity = 5.0 m/s (**b**) inlet velocity = 10.0 m/s (**c**) inlet velocity = 15.0 m/s (**d**) inlet velocity = 20.0 m/s.

Where *d* is the particle size of the dispersed phase; *ρ*_*o*_ is the density of the dispersed phase; *V*_*T*_ is the dispersed tangential velocity; *R* is the radial distance dispersed from the axis; *ρ*_*w*_ is the density of the continuous phase medium; *µ* is the dynamic viscosity of multiphase mixtures; *V*_*R*_ is the radial relative velocity between the dispersed phase and the continuous phase. After the structural dimensions of the hydrocyclone are fixed, as the inlet flow velocity increases from 5.0 m/s to 20.0 m/s, the radial pressure in the cone section shows an increasing trend. When the oil content of the mixture is 10%, the maximum radial pressure in the cone section is 10.37 kPa. When the inlet flow rate increases to 20 m/s, the maximum radial pressure is 57.50 kPa. At this point, the radial migration ability of the dispersed phase oil droplets in the mixed liquid increases, and the outflow channel of the oil phase medium changes from the overflow port and bottom flow port at low inlet flow velocity (5.0 m/s ~ 10.0 m/s), as shown in Fig. 6a and b, to the overflow port at high inlet flow velocity (15.0 m/s), as shown in Fig. 6c. Continuing to increase the inlet flow rate may also increase the radial migration ability of dispersed phase oil droplets, but it also increases the shear force, causing some degree of damage to the oil droplets. The radial force borne by oil droplets with smaller particle size is less than the sum of centrifugal force *Fn* and Stokes Resistance *Fs*, so they flow out from the bottom outlet, as shown in Fig. 6d. In addition, the density and viscosity of the dispersed phase medium in the mixed liquid are also important factors affecting the radial pressure distribution, as shown in Fig. 5a–d. At the same inlet flow rate, as the oil content of the mixture increases from 10% to 30%, the radial pressure shows a decreasing trend. This is mainly due to the increase in oil content in the mixed liquid, which leads to a decrease in dispersed phase density and an increase in viscosity. The centrifugal force *Fn* decreases to a certain extent, and the Stokes Resistance *Fs* increases. Therefore, the radial pressure also shows a decreasing trend.

### Turbulence level

**Fig. 7:**
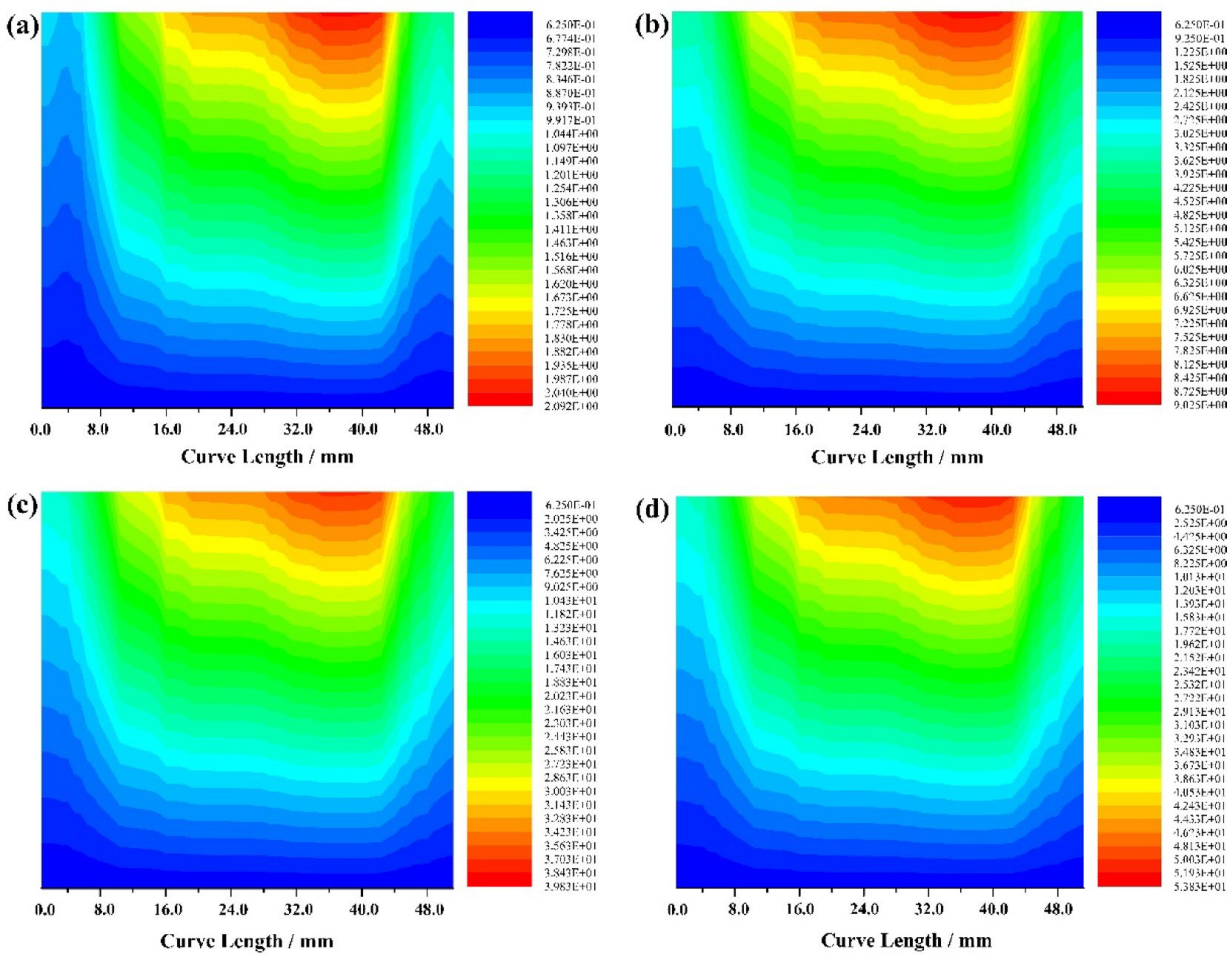
Contour lines of oil phase turbulent energy distribution in the cone section (**a**) inlet velocity = 5.0 m/s (**b**) inlet velocity = 10.0 m/s (**c**) inlet velocity = 15.0 m/s (**d**) inlet velocity = 20.0 m/s.

The turbulence intensity of the conical section of a hydrocyclone is a key parameter for measuring the flow stability of a mixed liquid fluid in the conical region. Its magnitude directly affects the oil-water separation efficiency of the mixed liquid and the distribution of dispersed phase oil droplet size. As shown in Fig. 7, when the oil content of the mixed liquid is 10%, the turbulence level obtained by setting monitoring curves at the cone section Y=-270 and X=-25 of the hydrocyclone. At different inlet flow velocities, the turbulence level of the mixed liquid in the cone section of the hydrocyclone shows a trend of forced vortices being higher than free vortices.

**Fig. 8:**
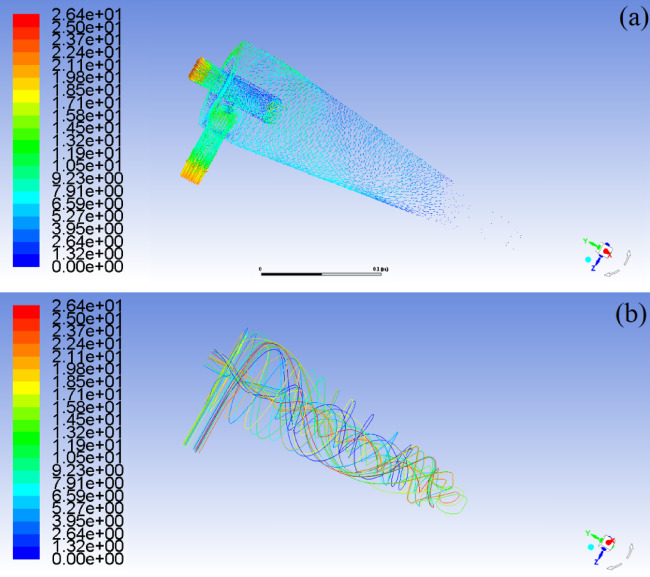
Vector diagram and streamline distribution of oil phase during hydrocyclone separation. (**a**) Vector diagram of oil phase (**b**) Streamline distribution of oil phase.

It is worth noting that when the oil content of the mixed liquid is 10% and the inlet flow velocity is 5.0 m/s, the maximum turbulent kinetic energy of the forced vortex zone is 2.09m^2^/s^2^. When the inlet velocity is 10.0 m/s, the maximum turbulent energy in the forced vortex zone is 9.05m^2^/s^2^, which is 4.33 times higher than when the inlet velocity is 5.0 m/s. When the inlet velocity is 15.0 m/s, the maximum turbulent energy in the forced vortex zone is 39.83m^2^/s^2^, which is 4.40 times higher than when the inlet velocity is 10.0 m/s. When the inlet velocity is 20.0 m/s, the maximum turbulent energy in the forced vortex zone is 53.83m^2^/s^2^, which is 1.35 times higher than when the inlet velocity is 15.0 m/s. This is because increasing the inlet velocity from 5.0 m/s to 15.0 m/s is beneficial for improving the turbulence intensity in the forced vortex zone of the cone section and increasing the coalescence of oil droplet size in the central area when the oil content does not change, as shown in Fig. 8a and b.

**Fig. 9:**
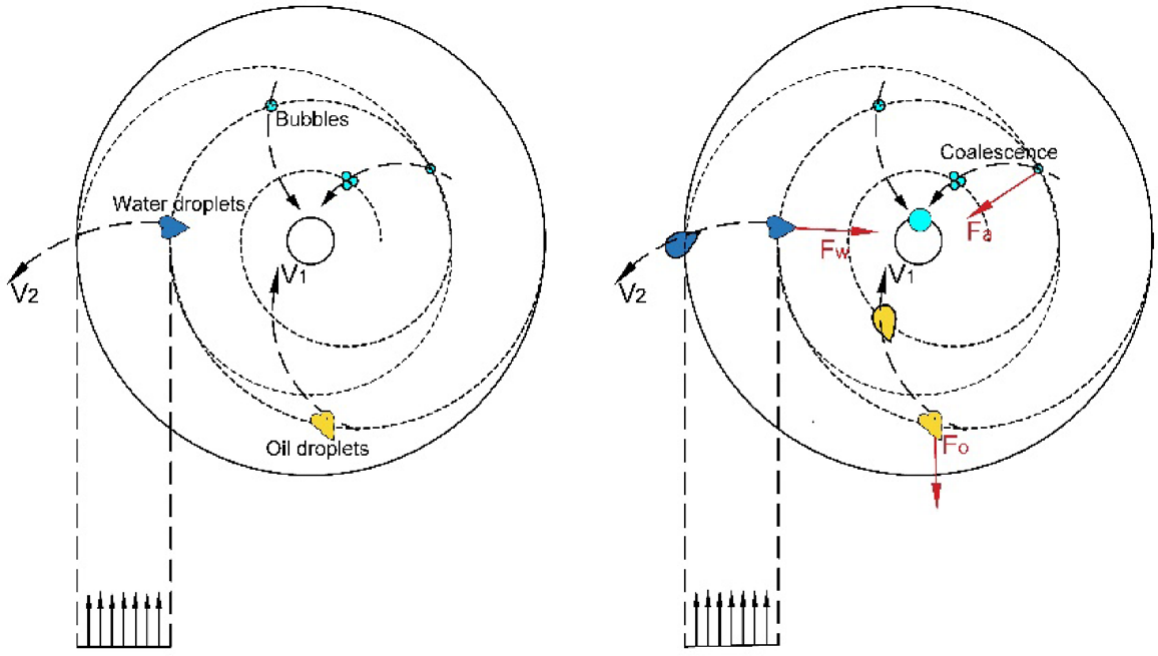
Flow field characteristics and droplet coalescence features of oil phase medium.

After entering the cyclone through the tangential feed inlet, the oil-water mixture undergoes high-speed rotational motion, and the flow field is divided into the outer quasi free vortex zone and the axial forced vortex zone. In the quasi free vortex region, the tangential velocity of the fluid increases as the radius decreases. After entering the forced vortex zone, the fluid particles rotate in a rigid body state, and the angular velocity tends to be consistent. According to the Bernoulli equation of the rotating flow field, the static pressure energy of the fluid will be converted into kinetic energy, resulting in a sharp decrease in the axial pressure of the forced vortex zone, even lower than the ambient atmospheric pressure. In oil-water mixtures, both water and oil dissolve a certain amount of air (such as air or hydrocarbon gases commonly dissolved in groundwater and crude oil). When the pressure in the forced vortex zone drops to the gas saturation and precipitation pressure at the operating temperature, a large amount of gas dissolved in the oil and water phases will precipitate.

If the pressure further drops to the saturated vapor pressure of water, the water will undergo local vaporization, and the generated water vapor will also enter the forced vortex zone. When the inlet flow velocity reaches 20.0 m/s, the increase in shear force will cause a change in the force on the water and oil phase media in the cone section of the hydrocyclone, as shown in Fig. 9. Whether it is the centripetal motion of oil phase media, gas phase media, or the centrifugal motion of water phase media. After coalescence, the droplets will be subjected to viscous drag force *Fa*. Under the combined action of viscous drag force and centrifugal force *Fw*, the radial flow of the mixed liquid in the cone section of the hydrocyclone will increase the resistance *Fo* along the way. However, during the process of increasing the inlet velocity of the mixed liquid from 15 m/s to 20 m/s, the maximum radial pressure in the cone section only increased from 45.59 kPa to 57.50 kPa. Due to insufficient resistance to shear stress, the probability of oil droplet coalescence is significantly higher than that of fragmentation, and the degree of increase in turbulence intensity in the cone section is reduced^37^.

### Oil phase volume fraction and droplet size distribution

**Fig. 10:**
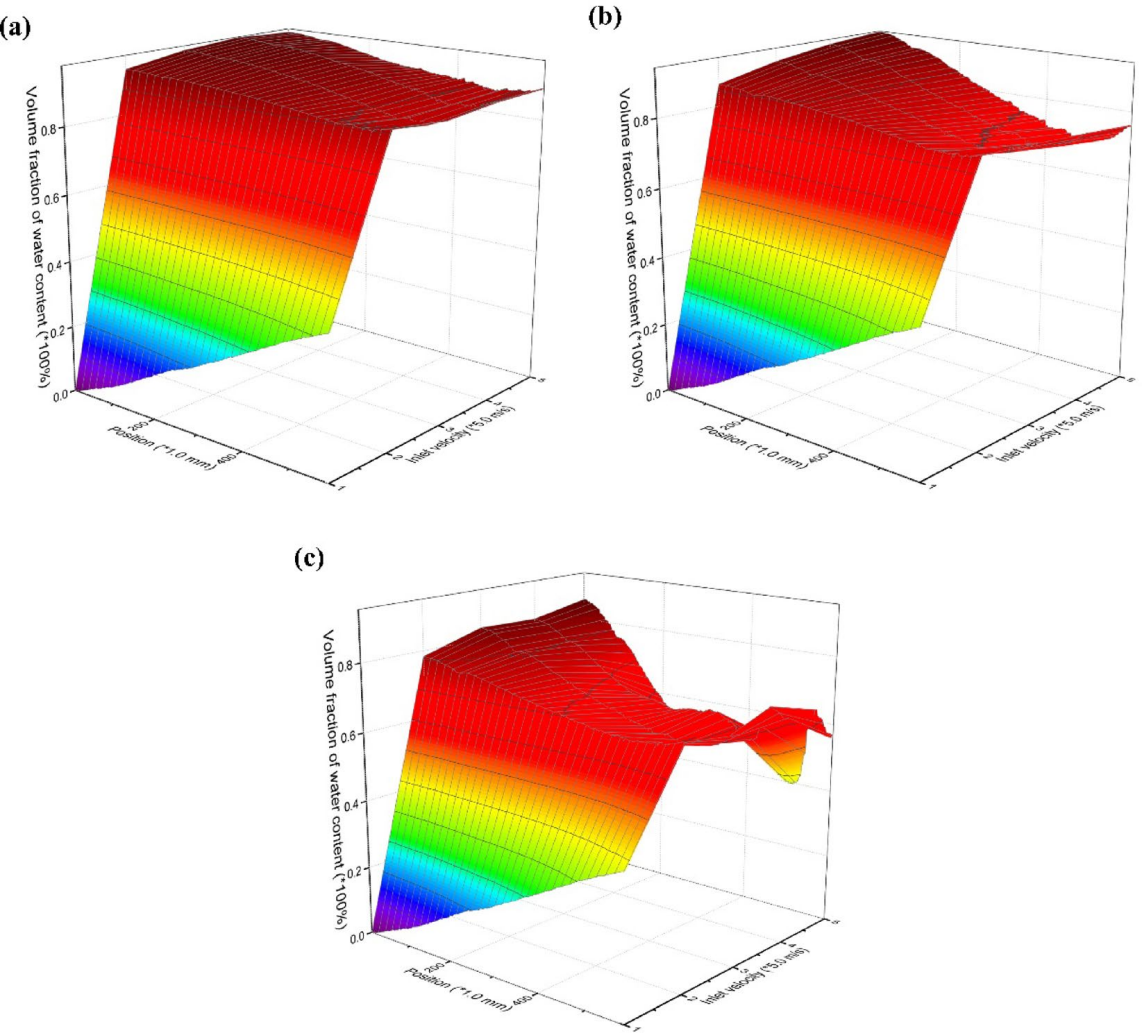
The influence of inlet velocity and oil content of mixed liquid on the axial distribution of oil phase. (**a**) The oil content is 10%, (**b**) The oil content is 20%, (**c**) The oil content is 30%.

A monitoring curve X=-25, Z = 0 is set from the overflow port to the bottom flow port of the hydrocyclone to obtain the distribution characteristics of the axial oil phase volume fraction in the forced vortex zone of the hydrocyclone as a function of the inlet flow velocity and oil volume fraction of the mixed liquid, as shown in Fig. 10. Under the unchanged structure of the hydrocyclone, the inlet flow velocity and viscosity of the mixed liquid will affect the distribution of the oil phase medium. At the same inlet flow rate, the oil phase medium in the mixed liquid is mainly distributed in the forced vortex zone of the hydrocyclone. The closer it is to the overflow port, the higher the volume fraction of the oil phase medium, and a small amount of oil phase medium flows out from the bottom flow port.

**Fig. 11:**
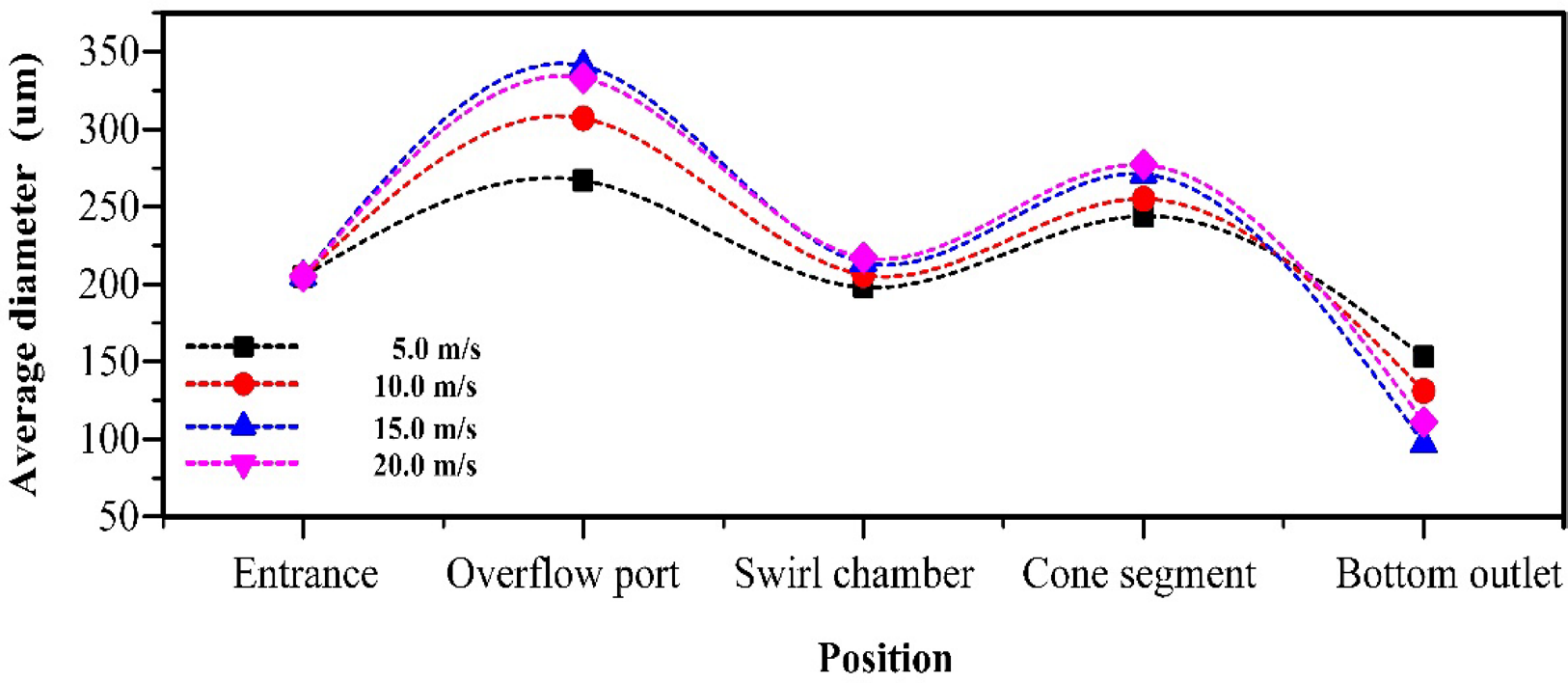
The influence of inlet velocity on the distribution of average droplet size when the oil content is 10%.

 When the oil content of the mixed liquid is 10%, as shown in Fig. 10a, the process of increasing the inlet flow velocity from 5.0 m/s to 15.0 m/s will enhance the centrifugal force field. This makes the distribution of oil phase more concentrated on the axis, and the probability of coalescence of dispersed phase medium is higher than that of fragmentation. At this point, when the inlet particle size of the oil phase medium is 200 μm, the droplet size of the overflow port, swirl chamber, and cone section all increase with the increase of the inlet flow velocity of the mixed liquid, as shown in Fig. 11. This also leads to changes in the flow field characteristics of the oil phase medium in the mixed liquid with increasing inlet velocity, reducing the oil volume fraction at the bottom outlet, as shown in Fig. 6a–c. However, as the inlet velocity continued to increase to 20.0 m/s, the increase in shear force led to an increase in the proportion of short circuiting flow of dispersed phase oil droplets, and the coalescence frequency of oil droplets was lower than the fragmentation frequency, resulting in a small amount of dispersed phase medium flowing out from the bottom outlet, as shown in Fig. 6d.

**Fig. 12:**
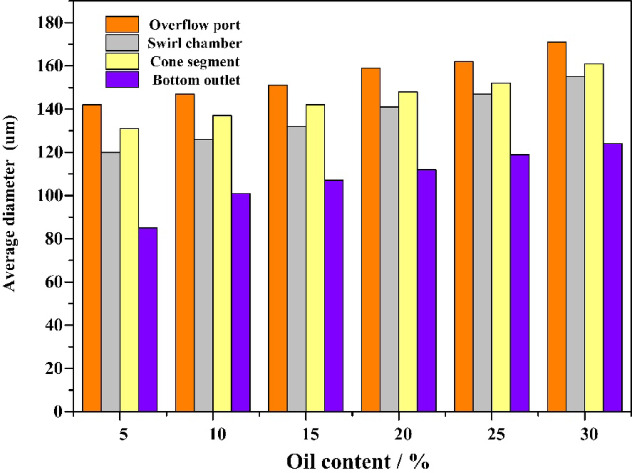
The average particle size distribution of oil phase medium droplets.

On the other hand, the oil content can also affect the volume fraction distribution characteristics of the dispersed phase medium by changing the viscosity of the mixture. The increase in oil content in the mixed solution will enhance the probability of aggregation of dispersed phases in the forced vortex zone, as shown in Fig. 12. When the inlet particle size of the oil phase medium is 100 μm, as the oil content of the mixed liquid increases, the dispersed phase droplets at the overflow port of the hydrocyclone, and the cone section all show an increasing trend. On the other hand, an increase in the oil content of the mixed liquid will lead to an increase in the Stokes resistance of the radial migration of dispersed phase oil droplets. Extend the residence time of oil droplets in the forced vortex zone. Increase the probability of shear failure of oil droplets. Therefore, the axial oil phase volume fraction of the hydrocyclone exhibits significant fluctuations, especially at the overflow port where the volume fraction decreases significantly, as shown in Fig. 10b and c. The higher the inlet flow rate, the higher the oil content in the mixed liquid, and the more severe the shear failure of oil droplets. As the volume fraction of oil in the bottom outlet increases, the particle size of oil droplets also increases. The control law of oil droplet particle size distribution and oil droplet morphology (dispersed/coalesced) in oily wastewater by cyclone pretreatment was clarified through simulation. Under the current structural parameters of hydrocyclones, they can effectively intercept oil droplets with a particle size greater than 80 µ m. At the same time, through the vortex shear and coalescence effect, some small oil droplets are coalesced into larger oil droplets, so that the particle size distribution of the pretreated water inlet oil droplets is concentrated in the range of 140–170 µ m. This elucidates that “the cyclone can intercept large oil droplets and regulate the coalescence of small oil droplets”, which can significantly reduce the deposition and blockage of large oil droplets on the membrane surface, while reducing the irreversible pollution risk caused by the infiltration of small oil droplets into the membrane pores.

## Conclusion

1. The distribution of oil phase volume fraction in a hydrocyclone is comprehensively controlled by inlet flow velocity, oil content, and structural parameters such as cone angle and overflow pipe depth. The radial flow velocity in the swirl chamber is generally M-shaped, and the tangential flow velocity near the wall in the free vortex region is higher than that in the forced vortex region. The tangential flow velocity in the cone section follows an axisymmetric distribution, and the tangential flow velocity in the forced vortex region increases with the radius, reaching a maximum value of about 1.75–1.87 times the inlet flow velocity.
2. At low to medium flow velocities (5.0 ~ 15.0 m/s), the centrifugal force field dominates the enrichment of oil towards the axis, and the coalescence effect is enhanced. At high inlet flow rates, the oil phase outflow channel changes from a dual channel of overflow and bottom flow to mainly overflow. When the inlet flow rate reaches 20 m/s, the shear force increases, causing the oil droplets to break and the radial migration path to deviate from the design. The flow velocity distribution becomes more complex, resulting in the distribution characteristics of the oil volume fraction in the mixed liquid becoming more complex, and small-sized oil droplets are discharged from the bottom flow.
3. Based on the simulation results, further analyze the influence of key parameters such as inlet flow rate, cone angle, and overflow ratio of the cyclone on the oil droplet control effect. Clarify the optimization range of cyclone pretreatment on membrane separation performance under different parameters, providing theoretical basis for engineering parameter optimization of cyclone membrane separation coupling system.
4. The increase in oil content enhances the probability of coalescence by increasing viscosity, but also increases Stokes resistance, resulting in prolonged droplet retention time, increased risk of shear failure, and intensified fluctuations in oil phase volume fraction at the overflow port. The oil content changes the viscosity of the fluid, promoting the coalescence of dispersed phase oil droplets while inhibiting migration. Efficient separation can be achieved by optimizing the balance between flow rate and oil content.

## Data Availability

All data generated or analysed during this study are included in this published article.
